# Disruption of Transporters Affiliated with Enantio-Pyochelin Biosynthesis Gene Cluster of *Pseudomonas protegens* Pf-5 Has Pleiotropic Effects

**DOI:** 10.1371/journal.pone.0159884

**Published:** 2016-07-21

**Authors:** Chee Kent Lim, Anahit Penesyan, Karl A. Hassan, Joyce E. Loper, Ian T. Paulsen

**Affiliations:** 1 Department of Chemistry and Biomolecular Sciences, Faculty of Science and Engineering, Macquarie University, Sydney, New South Wales, Australia; 2 USDA-ARS Horticultural Crops Research Laboratory and Department of Botany and Plant Pathology, Oregon State University, Corvallis, Oregon, United States of America; Centre National de la Recherche Scientifique, Aix-Marseille Université, FRANCE

## Abstract

*Pseudomonas protegens* Pf-5 (formerly *Pseudomonas fluorescens*) is a biocontrol bacterium that produces the siderophore enantio-pyochelin under conditions of iron starvation in a process that is often accompanied by the secretion of its biosynthesis intermediates, salicylic acid and dihydroaeruginoic acid. In this study, we investigated whether several transporters that are encoded by genes within or adjacent to the enantio-pyochelin biosynthetic cluster, serve as efflux systems for enantio-pyochelin and/or its intermediates. In addition, we determined whether these transporters have broad substrates range specificity using a Phenotype Microarray system. Intriguingly, knockouts of the *pchH* and *fetF* transporter genes resulted in mutant strains that secrete higher levels of enantio-pyochelin as well as its intermediates salicylic acid and dihydroaeruginoic acid. Analyses of these mutants did not indicate significant change in transcription of biosynthetic genes involved in enantio-pyochelin production. In contrast, the deletion mutant of PFL_3504 resulted in reduced transcription of the biosynthetic genes as well as decreased dihydroaeruginoic acid concentrations in the culture supernatant, which could either point to regulation of gene expression by the transporter or its role in dihydroaeruginoic acid transport. Disruption of each of the transporters resulted in altered stress and/or chemical resistance profile of Pf-5, which may reflect that these transporters could have specificity for rather a broad range of substrates.

## Introduction

Siderophores are iron-chelating compounds produced by many microorganisms to assist in the acquisition of iron from environments with low free-iron availability [1]. *Pseudomonas protegens* Pf-5 (previously known as *Pseudomonas fluorescens* Pf-5) is a soil bacterium and biocontrol agent that can suppress a wide variety of plant-pathogenic bacteria, fungi and oomycetes [2, 3]. Pf-5 produces siderophores from the pyoverdine (Pvd) and pyochelin (Pch) families, which differ structurally [4]. Further, their relative capacities to bind iron differ, with Pch displaying a lower iron-binding affinity than Pvd [5]. Production of Pch has been shown for many bacteria including *P*. *aeruginosa* [6], *Burkholderia cenocepacia* [7] and *Streptomyces scabies* [8]. Because of the lower affinity for iron of Pch, this siderophore could be considered to be secondary to Pvd. However, the biosynthesis of Pch may provide the benefit of conserving energy for the bacterium because Pvd biosynthesis requires more resources [9–11]. Therefore, it may be the iron acquisition system of choice when iron is not limited. Additionally, Pch may play other biological roles such as elicitation of induced systemic resistance in plants [12] or degradation of environmental toxic compounds, such as triphenyltin [13].

It was previously assumed that Pch produced by *P*. *aeruginosa* and *P*. *protegens* were identical [14–16] but a study by Youard *et al*. (2007) [17] showed that the siderophore from *P*. *protegens* Pf-5 is an optical antipode of the siderophore from *P*. *aeruginosa*, and is subsequently called enantio-pyochelin (E-Pch). As with the Pch of *P*. *aeruginosa* which is secreted as interconvertable diastereoisomers with 4’R,2”R,4”R (Pch I) and 4’R,2”S,4”R (Pch II) configurations [18], E-Pch secreted by *P*. *protegens* exists in two interconvertable diastereoisomers, *i*.*e*. E-Pch I (4’S, 2”S, 4”S) and E-Pch II (4’S, 2”R, 4”S) [17].

Due to their opposite alternate stereochemistry, Pch and E-Pch cannot be used interchangeably between the producing *Pseudomonas* species [17]. One reason for this is the stereospecificity of their cognate outer membrane uptake systems for the iron-loaded siderophore; the receptor FptA in *P*. *aeruginosa* is specific for iron-Pch complex while the receptor FetA in *P*. *protegens* is specific for the iron-E-Pch complex [19]. Uptake of the iron-complexed siderophores through the inner membrane into cytoplasm between the two *Pseudomonas* species is facilitated by different transporters, where iron-Pch might cross via the FptX permease in *P*. *aeruginosa* [20] while iron-E-Pch complex utilize the FetCDE permease in *P*. *protegens* [21].

**Fig 1:**
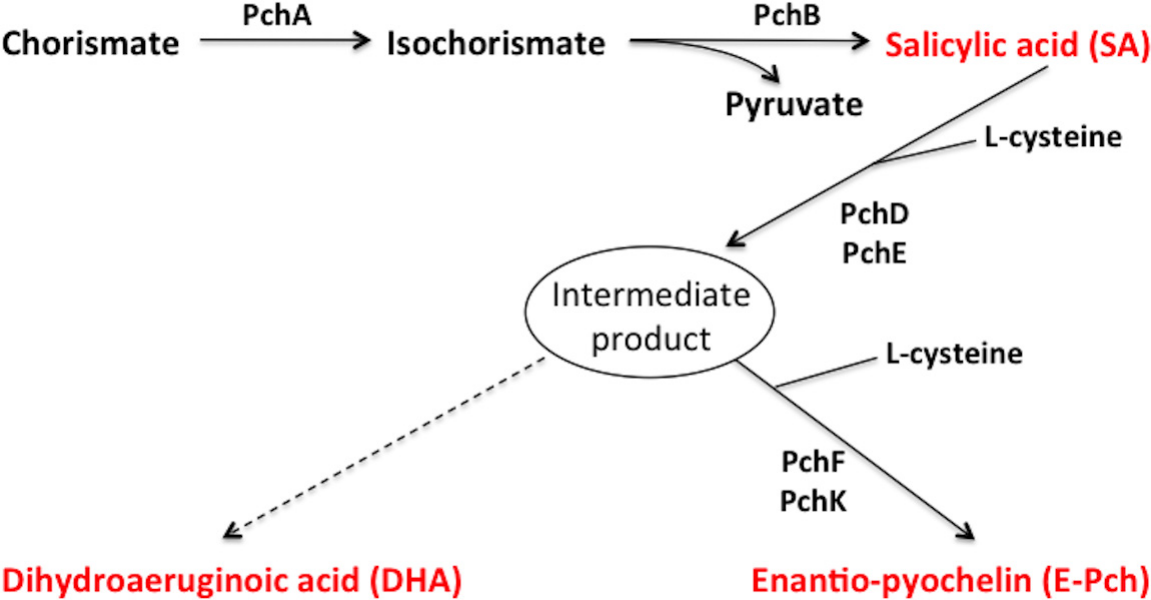
Pathway for biosynthesis of salicylic acid (SA), Dihydroaeruginoic acid (DHA) and Enantio-pyochelin (E-Pch), based on the information provided in Maspoli et al., 2014 [30].

In addition to the final Pch or E-Pch biosynthesis products, certain intermediates that are derived from the pathway, such as salicylic acid (SA) and dihydroaeruginoic acid (DHA) (Fig 1), can be exported out of the cell [22–25]. This raised a question as to whether these intermediates have extracellular roles that relate to the physiology of the producing organism. Indeed, SA can chelate iron and serve as a siderophore in its own right in *P*. *aeruginosa* and *P*. *fluorescens* [22, 26, 27]. Furthermore, SA can activate plant-induced systemic resistance against certain pathogens [28, 29]. Although DHA has the capacity to bind iron, it does not function predominantly as a siderophore [24]. DHA may have other natural functions as this compound was shown to have anti-fungal and anti-microbial properties [23], suggesting a role in antibiosis.

While the Pch uptake systems of *Pseudomonas* species are relatively well studied, the mode of export is less certain. An attempt to elucidate the role of PchHI ABC transporter in Pch secretion in *P*. *aeruginosa* demonstrated that null mutations of these genes did not affect Pch level in culture supernatant [31]. There is a possibility that multiple Pch secretion systems are present in *P*. *aeruginosa* [17], thus the PchHI null mutation phenotype may have been compensated by another transporter.

Within and adjacent to the characterized E-Pch biosynthetic gene cluster of *P*. *protegens* Pf-5, there are genes encoding putative inner membrane efflux transporters with unknown roles or substrate specificities. These include genes (PFL_3494 and PFL_3495) encoding an ABC transporter that is homologous to PchHI in *P*. *aeruginosa*, and Major Facilitator Superfamily (MFS) transporter genes *fetF* (PFL_3503) and PFL_3504. Recently, FetF in *P*. *protegens* CHA0 was determined not to be essential for iron-E-Pch complex uptake and is proposed to be involved in E-Pch recycling [21]. In this study, we characterized the roles of these putative transporters in E-Pch production. In addition, we found that mutations in each of the three putative transporters altered the sensitivity of Pf-5 to one or more antibiotics or other toxic compounds which could imply that these transporters have a broad substrate range.

## Materials and Methods

### Bioinformatics

Potential exporters for E-Pch that are encoded within or adjacent to the E-Pch biosynthetic gene cluster in *Pseudomonas protegens* Pf-5 (PFL_3488–3503) [17, 32] were identified using the Transporter Automated Annotation Pipeline [33]. Analysis of the transmembrane segments and topology was performed with HMMTOP version 2.0 transmembrane topology prediction server [34]. Conserved domains in the proteins were identified using Conserved Domain Database [35]. Information regarding prediction of operon structure was garnered from the Database of prOkaryotic OpeRons (DOOR) version 2.0 [36].

### Bacterial strains, plasmids and growth conditions

The bacterial strains and plasmids used in this study are shown in Table 1. The *Pseudomonas protegens* Pf-5 strain was used as the model for our study. *Escherichia coli* TOP10 was used for routine cloning procedures and propagated in Luria-Bertani (LB) medium. Biparental mating was performed on LB plates supplemented with 1% (W/V) glycerol, using the *E*. *coli* S17-1 strain as a donor. For extraction of E-Pch and its intermediates, *P*. *protegens* Pf-5 strains were grown in M9 minimal medium supplemented with 100 μM calcium chloride (CaCl_2_), 2 mM magnesium sulphate (MgSO_4_) and 0.4% glucose [37], at 25°C with shaking until OD_600_ reached approximately 0.8. Iron was not included in the media so as to promote siderophore production.

**Table 1:** Strains and plasmids used in this study.

Strains or plasmids	Genotype or characteristics	Sources
**Strains**		
*Pseudomonas protegens* Pf-5	Wild-type	Howell and Stipanovic (1979)
Δ*pchH*	*P*. *protegens* Pf-5 Δ*pchH*	This study
Δ*fetF*	*P*. *protegens* Pf-5 Δ*fetF*	This study
Δ3504	*P*. *protegens* Pf-5 ΔPFL_3504	This study
*Escherichia coli* S17-1	*recA thi pro*, *tra* genes (from plasmid RP4) integrated into chromosome	Simon *et al*. (1983)
*Escherichia coli* TOP10	F- *mcrA* (*mrr-hsdRMS-mcrBC*) *80lacZM15 lacX74 recA1 ara139* (*ara-leu*)*7697 galU galK rpsL* (Str^R^) *endA1 nupG*	Invitrogen
**Plasmids**		
pGEM-T Easy	TA cloning plasmid vector, Ap^R^	Promega
pEX18Tc	Broad-host-range suicide plasmid vector; Tc^R^	Hoang *et al*. (1998)
pEX18Tc-3495NF	1.1 kb PCR fragment containing truncated (from nt 254 to nt 1464) *pchH* gene cloned into *Hin*dIII-*Bam*HI sites of pEX18Tc; used to construct Δ*pchH* strain	This study
pEX18Tc-3503NF	1.1 kb PCR fragment containing truncated (from nt 121 to nt 920) *fetF* gene cloned into *Hin*dIII-*Bam*HI sites of pEX18Tc; used to construct Δ*fetF* strain	This study
pEX18Tc-3504NF	1.1 kb PCR fragment containing truncated (from nt 82 to nt 1011) PFL_3504 gene cloned into *Hin*dIII-*Bam*HI sites of pEX18Tc; used to construct Δ3504 strain	This study
pBBR1-MCS2	5.1-kb broad-host range plasmid; Km^R^ *lacZ*	Kovach *et al*. (1995)
pBBR1-pchH	Two distinct PCR products of *pchH* and native promoter region upstream of *pchD* separately cloned into pBBR1-MCS2 at *Hin*dIII-*Eco*RI and *Eco*RI-*Bam*HI sites respectively	This study
pBBR1-fetF	PCR product containing *fetF* gene with downstream terminator region was cloned into *Hin*dIII-*Eco*RI site of pBBR1-MCS2 while a PCR product containing the native promoter region upstream of *fetA* was cloned into the same pBBR1-MCS2 vector at *EcoRI*-*Bam*HI site	This study
pBBR1-3504	PCR product containing contiguous PFL_3504 gene with native promoter and terminator regions cloned into pBBR1-MCS2 at *Hin*dIII-*Eco*RI sites.	This study

### Construction of knockout mutants

In this study, deletion of the *pchH*, *fetF* and PFL_3504 genes was performed via an allelic exchange procedure adapted from Hoang *et al*. (1998). The truncated genes used to generate deletions within the chromosomal genes were constructed using either KOD Hot Start DNA Polymerase (Merck) or GC-RICH PCR System (Roche), employing the splicing overlap extension (SOE) PCR technique adapted from Choi and Schweizer (2005) [38]. Primers used for mutagenesis are listed in S1 Table. Briefly, the regions around the 5’ and 3’ ends of the genes of interest were PCR amplified and spliced together using SOE PCR method. Cloning of the truncated gene into suicide vector pEX18Tc was performed at *Hin*dIII and *Bam*HI sites through restriction digestion and ligation, thus creating the recombinant vectors with the truncated genes.

The recombinant suicide vector containing the truncated gene was chemically transformed into *E*. *coli* S17-1. Biparental mating was performed between S17-1 carrying the recombinant plasmid and wild-type (WT) Pf-5 in order to mobilize the plasmid into Pf-5 through conjugation. Briefly, cells were mixed in 1:1 ratio (based on OD_600_) and plated onto LB agar containing 1% glycerol (W/V) and incubated for 6 hours at 37°C. After the incubation, cells were harvested from the plate, resuspended in 5 mL 0.1 M MgSO_4_, and 100 μl of the cell suspension was spread on LB agar medium containing 200 μg/mL tetracycline and 100 μg/mL streptomycin to select for Pf-5 bacteria (Pf5 has innate resistance against streptomycin) carrying the recombinant plasmids. The surviving colonies were propagated in 1 mL of non-selective LB media at 25°C for 2–3 hours and plated onto LB agar containing 5% sucrose. The high sucrose content selected against bacteria that carried pEX18Tc due to the presence of *sacB* gene in the plasmid [39]. Colonies that survived the sucrose selection were patched in parallel onto LB agar containing 5% sucrose and LB agar containing 200 μg/mL tetracycline, the latter to confirm the absence of the recombinant pEX18Tc plasmid in the bacteria. The colonies were then screened by colony PCR with FastStart Taq DNA polymerase (Roche) and gene-specific primers (S2 Table) for mutants that have truncated genes in the chromosomes.

### Construction of plasmids for gene complementation

Gene sequences for complementation of deletion mutations were amplified by PCR using Pwo DNA polymerase (Roche). Primers used for PCR amplification are listed in S3 Table. All genes were cloned under the control of their putative native promoters. The *pchH* and *fetF* genes are located in predicted operons and are encoded distally to their predicted promoter sequence. Therefore, the promoter sequences controlling expression of their respective operons were amplified separately and cloned upstream of the genes in plasmid constructs for use in complementation. In constructing the gene complementation plasmid for the Δ*pchH* mutant, the region upstream of *pchD* (PFL_3496) (375 bp) was first cloned into pBBR1-MCS2 at *Hin*dIII and *Eco*RI sites. Subsequently, *pchH* was cloned into the *Eco*RI and *Bam*HI sites, creating pBBR1-pchH. The GTG start codon of *pchH* was changed to an ATG start codon in the cloned gene during PCR amplification to facilitate a more efficient translation. Similarly, in construction of the complementary plasmid for the Δ*fetF* mutant, the 177 bp region upstream of *fetA* was cloned into pBBR1-MCS2 at *Hin*dIII and *Eco*RI sites. The *fetF* gene, together with its 195 bp downstream region, was then cloned into the *Eco*RI and *Bam*HI sites of the previous plasmid construct to make the pBBR1-fetF plasmid. Lastly, the PFL_3504 gene, together with its upstream (134 bp) and downstream (188 bp) regions, was amplified and directly cloned into pBBR1-MCS2 at the *Hin*dIII and *Eco*RI sites. The integrity of the cloned genes, as well as promoter and terminator regions, was verified by DNA sequencing. The resulting complementation plasmids were transformed into *E*. *coli* S17-1 and then mobilized into the mutant strains via biparental mating as described above. Pf-5 WT strains carrying these plasmids (and the control plasmid pBBR1-MCS2) were maintained with 50 μg/ml kanamycin.

### Compound secretion profiling using High Performance Liquid Chromatography (HPLC)

Five mL of supernatant from spent cultures of Pf-5 WT and derivative strains were extracted to quantify E-Pch and biosynthetic intermediates. Spent media from three replicate cultures of each strain were acidified to pH 1–2 using hydrochloric acid and extracted twice with 2 mL of ethyl acetate. The extracts were dried under reduced pressure using a SpeedVac DNA 110 (Savant) and dissolved in 250 μL of methanol. Chemical analysis was performed using a Shimadzu HPLC instrument equipped with SPD-M10A diode array detector (Shimadzu) and Phenomenex Luna C18(2) column (4.6 x 250 mm, 5 μm). The conditions of HPLC analysis were adapted from Reimmann *et al*. (1998) [25]. A binary gradient consisting of solvent A (10 mM H_3_PO_4_) and solvent B (95% methanol in 10 mM H_3_PO_4_) was used as follows: 0–29 min with 20–83% solvent B followed by 7 min with 83–100% solvent B. Elution was carried out at 40°C with a flow rate of 1 ml/min. SA, DHA as well as E-Pch I and E-Pch II were eluted at 19.9, 22.8, 27.8 and 28.7 minutes respectively. Concentrations of metabolites were determined by comparison with purified standards. Two-tailed Student’s *t*-test was used for statistical analysis where P<0.05 was considered as significant.

### RNA extraction

The bacterial pellets used for RNA extraction were obtained from the same cultures used for HPLC analysis described above. Trizol (Invitrogen) and the RNAeasy kit (Qiagen) were used for extraction of RNA. Off-column RNAse-free DNAse (Ambion) treatments of the RNA samples were performed. Concentrations of RNA were determined using a Nanodrop ND1000 spectrophotometer (NanoDrop Technologies).

### Transcriptional analysis with qRT-PCR

Changes to the level of transcripts for genes in the E-Pch biosynthetic gene cluster were determined using quantitative reverse-transcriptase polymerase chain reaction (qRT-PCR). Gene-specific primers utilized in this study were designed with Primer3 software [40] (S4 Table). Our previous transcriptomic study on the effect of iron limitation on Pf-5 [41] determined that the transcript abundance of PFL_5586, which encodes ribosomal protein S7, was not affected by iron. Therefore, this gene was selected as an internal control in the current qRT-PCR experiments. The cDNA templates for the qRT-PCR experiments were made from RNA samples using the QuantiTect Reverse Transcription Kit (Qiagen). GoTaq® qPCR Master Mix (Promega) was used in the qRT-PCR reactions, which were done in a Mastercycler ep Realplex^4^ S (Eppendorf). Initial data processing was performed using Eppendorf Mastercycler ep Realplex 2.2 software and fold-changes were calculated using the method described by Pfaffl (2001) [42]. Duplicate qRT-PCR reactions were performed for each of three replicate cultures of each strain.

### Evaluation of pyoverdine production

Relative levels of Pvd in spent media from three replicate cultures of each strain were determined by measuring the absorbance of culture supernatants at 403 nm [43] using a DU640 spectrophotometer (Beckman Coulter, Fullerton, CA).

### Examination of stress and chemical resistance with phenotype microarrays

Profiling of stress and chemical resistance of the *P*. *protegens* strains used in this study was performed using Phenotype Microarrays (PM) (Biolog, Inc. Hayward, California, USA) with PM09-PM20 panels, in accordance with the manufacturer’s protocols. The panels PM09 and PM10 test for osmotic and pH stress respectively while panels from PM11 to PM20 test for sensitivity to 240 different antimicrobials. After the inoculations plates were then incubated in OmniLog automated plate reader at 25°C. A change in the dye colour due to reduction of the tetrazolium dye as a result of bacterial respiration was recorded at 15 minutes intervals for 72 hours. The kinetic data obtained was analyzed with OmniLog PM software (Biolog) and areas under the kinetic curves for WT strain and mutants were compared.

### Determining minimal inhibitory concentrations to confirm results from phenotype microarrays

Minimal inhibitory concentrations (MICs) were determined to confirm the chemical sensitivities of the mutant strains Δ*pchH*, Δ*fetF*, and Δ3504 where they differed from WT Pf-5 in the phenotype microarrays. Briefly, the WT and the mutants were inoculated into the wells of 96-well microtiter plates containing Mueller-Hinton (MH) broth and various concentrations of the compounds of interest. Three biological replicates were used in the experiment. Plates were incubated at 25°C for 24 hours with shaking and the OD_595_ was measured using the Multiskan EX plate reader (Thermo Electron Corporation, Waltham, MA). MH medium with no bacterial inoculum, also present in the wells of microtiter plates, was used as a reference; MH broth inoculated with bacteria with no compound of interest added was used as a positive control.

## Results

### Bioinformatic analysis of putative transporter genes

Using TransAAP [33] we identified several genes encoding transporters around the E-Pch biosynthetic gene cluster in the *P*. *protegens* Pf-5 genome [32]. The genes *pchH* (PFL_3495), *fetF* (PFL_3503) and PFL_3504 were selected for our study.

The predicted amino acid sequence of PchH of *P*. *protegens* Pf-5 is 43% identical to PchH of *P*. *aeruginosa* PAO1. The C-terminal region of the peptide sequence contains characteristic Walker A and B motifs that are involved in ATP binding and hydrolysis. In the N-terminal region, six helices that could be involved in transmembrane spanning were identified (S1 Fig). The encoded protein PchH has an architecture characteristic of a bacterial ABC transporter involved in export, *i*.*e*., the membrane-spanning domain and the ATP-binding domain are fused into a single polypeptide and the transmembrane region of each protomer contains six predicted transmembrane helices [44, 45]. The putative export function of this ABC transporter is also supported by the lack of a gene encoding periplasmic binding protein co-localizing with the cluster [44]. PchH may function with PchI, another distinct ABC transporter component, to form a complete heterodimeric transporter [31].

*fetF* and PFL_3504 code for transporters classified within the MFS of transport proteins and, according to HMMTOP predictions [34], both contain 12 transmembrane segments (S1 Fig). Recently, FetF was shown by Reimmann (2012) [21] to not be required for iron-E-Pch complex uptake in *P*. *protegens* CHA0 and was proposed to be involved in recycling of E-Pch.

The three transporter genes (*pchH*, *fetF* and PFL_3504) are located in different predicted operons. *pchH* resides in the *pchDHIEFKCBA* putative operon (PFL_3488–3496) while *fetF* resides in the *fetABCDEF* putative operon (PFL_3498–3503) [17]. PFL_3504 may be transcribed as a single mRNA, according to the DOOR database [36].

### Effects of *pchH*, *fetF* and PFL_3504 gene disruptions on the accumulation of E-Pch and its intermediates SA and DHA in growth media

To elucidate the possible roles of *pchH*, *fetF* and PFL_3504 in the efflux of E-Pch and its biosynthetic intermediates SA and DHA from *P*. *protegens* Pf-5, allelic exchange mutagenesis was performed (Table 1). Successful truncation of the genes was verified by PCR (S2 Fig). For complementation studies, wild type *pchH*, *fetF* and PFL_3504 were cloned downstream of their native promoters into the vector pBBR1-MCS2 and transferred into their respective Pf-5 knockout strains by conjugation.

HPLC analyses of extracts of supernatants from cultures of each transporter gene knockout mutant were undertaken to assay the levels of the secreted metabolites E-Pch I, E-PCh II, SA and DHA. In all assays, the levels of both E-Pch I and E-Pch II detected in the supernatants from the bacterial samples were very similar (data not shown). Given that E-Pch I and E-Pch II are the same siderophores that exist as interconvertable diastereoisomers [46], for comparative purposes, we pooled their levels and refer singularly to them as E-Pch.

**Fig 2:**
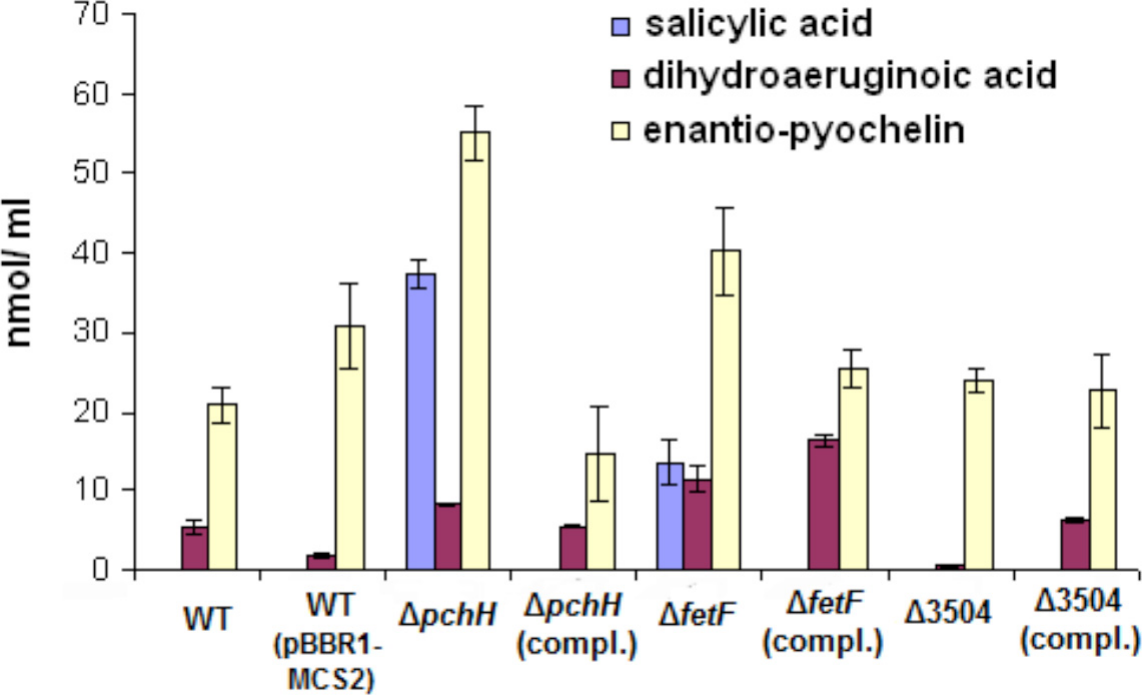
Concentrations of E-Pch, DHA and SA in culture supernatants of *P*. *protegens* strains determined by HPLC. Levels of E-Pch presented are combination of both E-Pch diastereoisomers. Error bars represent standard deviations between three biological replicates. Strains tested were *P*. *protegens* Pf-5 (wildtype) and mutants of Pf-5 having deletions in putative transporter genes (Δ*pchH*, Δ*fetF*, and Δ3504) as well as mutants complemented (labeled as ‘compl.’) with the corresponding gene in the plasmid vector pBBR1-MCS2. The label ‘WT (pBBR1-MCS2)’ indicates wild-type strain with control plasmid.

The levels of E-Pch and DHA were approximately 2.6- and 1.6-fold higher, respectively, in the Δ*pchH* mutant supernatants compared to the WT (*P* = 0.0001 and *P* = 0.007 respectively) (Fig 2). The SA concentration was below the HPLC detection limit in the supernatants from the WT cultures, but SA was found in abundance in the supernatants of the Δ*pchH* mutant. Complementation of this mutant strain with pBBR1-pchH restored E-Pch, SA and DHA production closer to WT levels (Fig 2).

E-Pch and DHA levels were also higher (1.9- and 2.1-fold, respectively) in supernatants of the Δ*fetF* mutant strain as compared to WT (*P* = 0.005 and *P* = 0.006 respectively) (Fig 2). Similar to strain Δ*pchH*, a substantial amount of SA was detected in culture supernatants of Δ*fetF*, which contrasted with the non-detectable levels in the WT supernatants. When compared to the Δ*pchH* mutant, supernatants of Δ*fetF* had lower SA levels and slightly higher DHA levels. Gene complementation of the Δ*fetF* mutant using pBBR1-fetF plasmid resulted in the cessation of SA secretion into the supernatant but, oddly, DHA level detected was slightly higher in complemented Δ*fetF* mutant than the mutant strain alone (*P = 0*.*01*).

The PFL_3504 gene truncation resulted in a 10.1-fold reduction in the DHA level in the supernatant in comparison to the WT (*P* = 0.001). Complementation with pBBR1-3504 reverted this Δ3504 phenotype to the WT phenotype (Fig 2). This suggests a potential role of PFL_3504 in DHA secretion.

### Effects of *pchH*, *fetF* and PFL_3504 gene disruptions on the transcription of the E-Pch biosynthetic gene cluster

**Fig 3:**
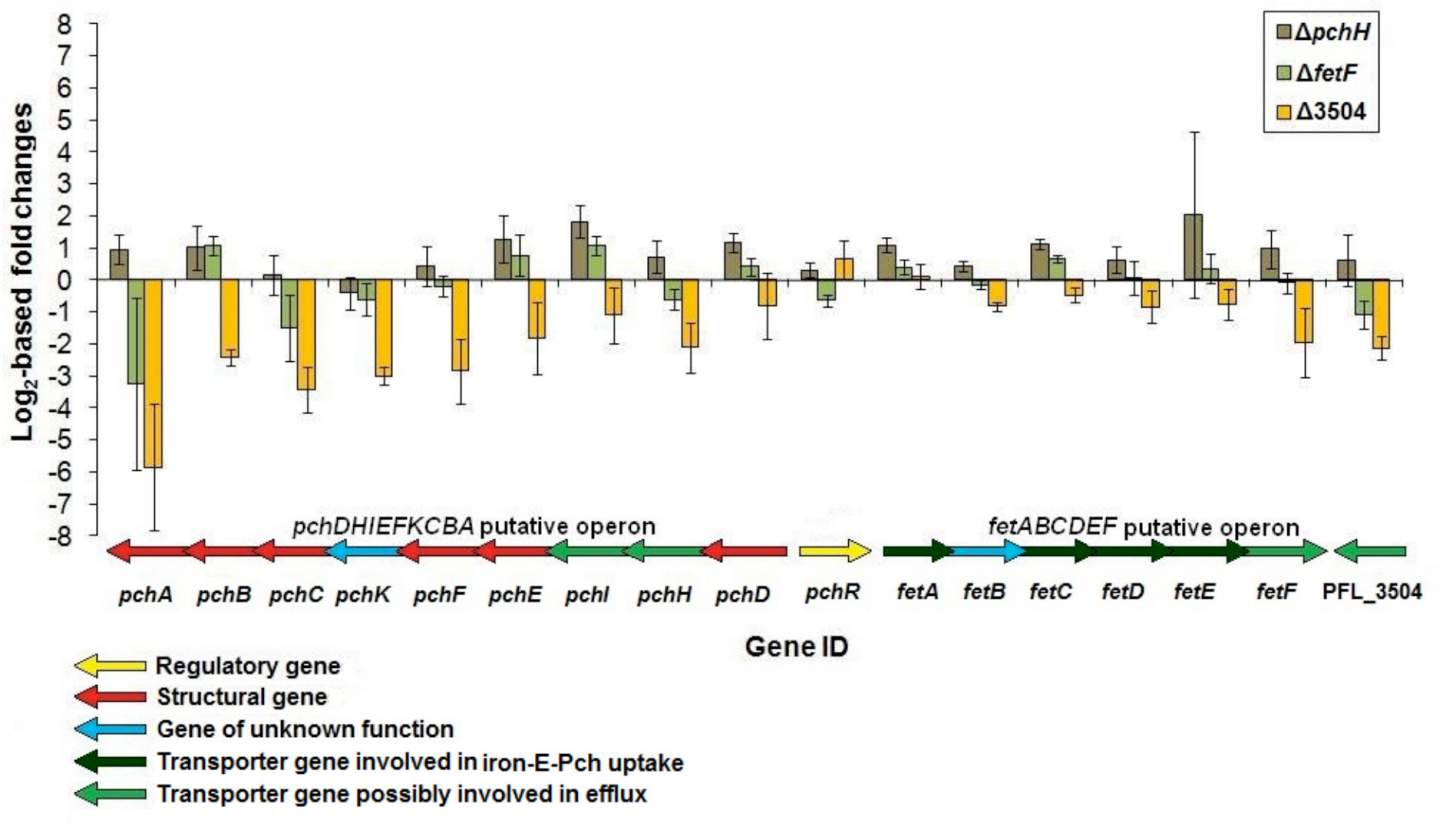
Transcriptional profiling of genes located around the enantio-pyochelin biosynthetic gene cluster of *P*. *protegens* Pf-5. Transcript levels were determined by qRT-PCR and the fold change (log_2_) of each transcript is shown for strains Δ*pchH*, Δ*fetF* and Δ3504 relative to WT Pf-5. qRT-PCR on the truncated genes was performed on non-truncated gene regions. Error bars represent standard deviations between replicates. Coloured arrows depict the corresponding gene organization with the putative operons of the E-Pch biosynthetic gene cluster.

qRT-PCR was used to determine whether the disruption of these putative transporter genes had any effect on the transcription of genes within the E-Pch biosynthetic gene cluster. The transcriptional profile for the Δ*pchH* and Δ*fetF* mutant strains did not show great differential transcription of the biosynthetic gene cluster when compared to the WT (Fig 3). Although a few genes within the cluster were highly transcribed, we surmised these are anomalies due to their large standard deviations (presumably because of experimental replicate variability) and their residence in possible polycistronic operons.

We observed reduced transcript levels of genes within *pchDHIEFKCBA* operon in the Δ3504 mutant strain when compared to the WT (Fig 3). Despite this, we did not observe a significant divergence between E-Pch levels in the supernatants from the Δ3504 strain versus the WT, although lower DHA concentrations were detected in the supernatants of the mutant strain.

To preclude the possibility that the changes in E-Pch levels observed are due to the variations in iron availability between the WT and mutants, we also analyzed the secreted Pvd levels in the supernatants of the same cultures. No notable differences in the Pvd levels from the different supernatants were found (S3 Fig), implying that difference in the availability of iron between the samples are not likely to significantly contribute to the observed differences in E-Pch production.

### Effects of transporter gene disruptions on stress and chemical resistance of mutant strains

As efflux pumps can have broad substrate specificities, we investigated whether these transporters play a role in stress and chemical resistance in Pf-5. This was examined using Biolog PM technology. Selected observations obtained from the Biolog PM study were confirmed in independent tests. Several alterations in stress and chemical resistance were observed between the WT and the mutant strains (S4 Fig). One of the most profound effects observed was the increased sensitivity of the Δ3504 strain to rifamycin SV (S4 Fig). Subsequently, we found that all of the transporter mutants were more susceptible to this antibiotic compared to the WT (Table 2). Conversely, mutations in some of the transporters resulted in increased resistance to chemical stress (S4 Fig; Table 2). One example is the increased resistance of all three mutants against dichlofluanid (Table 2). In some other cases, observations derived from PM studies could not be verified by independent tests. For example, we observed that the Δ*fetF* and Δ3504 strains were more sensitive to 1-chloro-2,4-dinitrobenzene in the PM experiments (S4 Fig) but they were found to display similar susceptibilities to this chemical as the WT strain when examined using the independent confirmatory test (Table 2).

**Table 2:** Sensitivity of Pf-5 and derivative strains to antibiotics and other compounds.

Chemicals	PM plate (well positions)	Wild-type		Δ*pchH*		Δ*fetF*		Δ3504	
		PM	MIC (μg/ml)	PM	MIC (μg/ml)	PM	MIC (μg/ml)	PM	MIC (μg/ml)
Phenylethylamine (in %, V/V)	PM10 (G8)	S	0.125	R	**0.25**	S	0.125	S^**!**^	**0.25**
9-Aminoacridinehydrochloride monohydrate	PM14 (B3, B4)	S	125	S	124	S^**!**^	**500**	R	**500**
Domiphen bromide	PM15B (D8)	S	15	M	15	R	**30**	R	**60**
1-Chloro-2,4-dinitrobenzene	PM16A (D3)	R	60	R	60	S^**!**^	60	S^**!**^	60
Dichlofluanid	PM16A (C3)	S	5000	R	**> 5000**	R	**>5000**	R	**>5000**
Rifamycin SV sodium salt	PM16A (E10, E11)	R	125	R^**!**^	**62.5**	R^**!**^	**62.5**	S	**62.5**
Cetylpyridinium chloride	PM16A (C3)	S	30	R^**!**^	30	R^**!**^	30	R	**60^**
Coumarin	PM19 (A11)	S	>1000	R	>1000	R	>1000	R	>1000
Phenylmethanesulfonyl fluoride	PM19 (D12)	S	>200	R	>200	R	>200	R	>200
Tolylfluanid*	PM20B (H5, H6, H8)	R	5000	S^**!**^	**> 5000**	S^**!**^	**>5000**	S^**!**^	**>5000**
Atropine	PM20B (C7)	M	>2000	M	>2000	R	>2000	R	>2000

Phenotypes observed in phenotypic microarray (PM) experiments (S: sensitive; R: resistant; M: moderately resistant) (S4 Fig), and independent tests determining minimal inhibitory concentrations (MICs). MICs were estimated from microdilution susceptibility assays of cultures grown in Mueller-Hinton broth. Phenotypes of mutant strains that differ from the wild-type are bolded.

* In the PM experiment, growth kinetics for the tested strains fluctuated across the four wells containing different concentrations of tolylfluanid (S4 Fig; PM20 H05-H08).

^**!**^ Discrepant phenotypes observed between PM experiments and independent confirmatory tests. Plausible reasons for these are given in text.

^ Confirmatory test was not consistent, as only one of three replicate cultures of Δ3504 exhibited resistance to cetylpyridinium chloride.

## Discussion

In this study, gene truncations were performed on the *pchH*, *fetF* and PFL_3504 transporter genes affiliated with E-Pch biosynthesis gene cluster in *P*. *protegens* Pf-5. We observed higher levels of E-Pch, SA and DHA in supernatants produced by both Δ*pchH* and Δ*fetF* mutants compared to the WT. This contradicted the expectation of lower levels of these compounds if these transporters were the primary efflux systems. Complementation of these mutants with intact genes driven by native promoters in pBBR1-MCS2 plasmids successfully reverted their phenotypes to resemble those of the WT. Specifically, no detectable levels of secreted SA were obtained from the complemented Δ*pchH* and Δ*fetF* mutants whereas secreted levels of E-Pch were reduced to levels similar to that of the WT. We observed low levels of DHA in the supernatant of the Δ3504 mutant where complementation of this mutation restored the DHA level in the supernatant to a level similar to that of the WT strain. The successful complementation experiments also implied that the increased production of the metabolites of interests was not due to polar effects as a consequence of mutagenesis.

The phenotype we observed for the *pchH* deletion mutant differed from a previous study examining the *P*. *aeruginosa pchH* homologue, where no significant change in Pch secretion was observed between a *pchH* deletion strain and the parental strain [31]. There is a possibility that the homologous ABC transporters between these two species have diverged to serve different functions. One possibility is to accommodate for the enantiomerism aspects of the siderophore cousins due to the notion that uptake of the enantiomers across the inner membrane occurs through entirely different types of transporters [17]. In addition, *pchH* of *P*. *aeruginosa* is implicated as a virulence factor in a few model species, where the virulence attenuation of the *pchH* mutant was apparently not related to iron-acquisition by pyochelin [47–49]. This possibly point to the distinct roles of this transporter in *P*. *protegens* and *P*. *aeruginosa*.

None of the three putative efflux systems targeted in this study was conclusively found to play a role in the export of E-Pch. This could be due to the presence of multiple functionally redundant exporters for this compound. Occurrences of redundant exporters for the same product are quite common in microbes. An example is the redundant transport functions of Hyg19 and Hyg28 transporters in the exportation of hygromycin A from *Streptomyces hygroscopicus*, albeit with different efficiencies [50]. Imperi et al. (2009) also showed the possible existence of multiple secretion systems of pyoverdine in *P*. *aeruginosa* [51].

To elucidate whether altered transcription of genes within the E-Pch biosynthetic gene cluster might be responsible for the increased production of the metabolites of interest, we performed transcriptional analyses on these genes. Our experiments indicated that no drastic change in transcription of these genes occurred in either Δ*pchH* and Δ*fetF* mutant strains with respect to wild-type but truncation of PFL_3504 resulted in reduced transcription of the *pchDHIEFKCBA* putative operon which might indicate that the transporter confers positive transcriptional regulation on the operon. While knockouts of the *pchH* and *fetF* genes do not significantly affect transcription of the biosynthetic gene cluster, they result in substantially higher levels of secreted salicylic acid, DHA and E-Pch. The reasons for this are unclear, but the knockout mutants may impact on translational or post-translational regulation of biosynthesis, or the availability of salicylic acid precursors

To test whether these transporters have the ability to transport a broad range of substrates, we performed a high-throughput experiment utilizing Biolog PM technology. These experiments demonstrated that mutations in the transporter genes altered the resistance of the bacteria towards certain stresses or chemicals (S4 Fig). Interesting phenotypes observed from these experiments were supported by microdilution susceptibility assays as an independent confirmatory test although some observational differences between the Biolog PM experiments and the independent tests were observed. Such discrepancies are not unusual, as seen in other studies [52, 53], and could be attributed to the experiment-to-experiment variation or differences in culture media used between the experiments which could affect antibiotic activities [54] as well as different detection approaches between the methods. To highlight the latter case, the Biolog PM system measures bacterial respiration whereas our confirmatory tests evaluated cell growth [55].

Nevertheless, it is very interesting to observe that knockouts of the transporters located within a siderophore biosynthetic cluster altered the stress and chemical resistance profile of the bacteria. Many efflux pumps are known to have promiscuous substrate specificities and are capable of pumping out a broad range of substrates rather than a single substrate [56]. Besides having a physiological role in the export of cellular products, some transporters that are associated with biosynthetic machinery are able to confer resistance against antibiotics such as the spermidine transporter Blt of *Bacillus subtilis* [57] that confers resistance to a variety of antibiotics [58]. Based on either PM experiments or the independent tests, we observed that knockouts of some of the transporters in our study increased the susceptibility of the mutants to certain antibiotics such as increased susceptibility to rifamycin SV in all three mutants in relative to WT (Table 2). Assays of the phenotypes of the complemented mutants showed reversion of resistance phenotypes (data not shown)

The transporter mutants were also found to be more resistant against certain stresses or antibiotics in comparison to the WT. For example, independent tests showed that the three mutant strains were more resistant toward the fungicides tolylfluanid and dichlofluanid than the WT strain. The resistance patterns for tolylfluanid and dichlofluanid, as determined by the independent tests, were similar between the strains which could be due to the structural similarity of these compounds [59]. Another example is the 4-fold increased resistance toward 9-Aminoacridine hydrochloride monohydrate by Δ*fetF* and Δ3504 strains (Table 2). Such increased antibiotic resistance as a result of transporter deletion is not unprecedented. In *Salmonella enterica*, disruption of the drug efflux pump AcrF resulted in increased resistance to a few antibiotics (Bailey *et al*., 2008) which could be attributed to compensatory increased expression of another drug efflux pump, AcrB [60]. Complementation of the ΔfetF and Δ3504 strains restored susceptibility toward 9- Aminoacridine hydrochloride monohydrate to the WT levels (data not shown). Interestingly, transposon-inactivation of *pchH* in *P*. *aeruginosa* genome resulted in decreased susceptibility to ciprofloxacin [61]. However, difference in susceptibility to ciprofloxacin was not observed between Pf-5 WT and the mutant strains in the PM experiments performed in this study.

In conclusion, our study examined the effect of *pchH*, *fetF* and PFL_3504 truncation in *P*. *protegens* Pf-5, with respect to the levels of secreted E-Pch and its biosynthetic intermediates SA and DHA. We found that these genes are not essential for the export of E-Pch or SA into culture supernatants. Conversely, the knockouts of *pchH* and *fetF* resulted in increased detection of the metabolites from the E-Pch biosynthetic locus. However, the mechanism responsible for this phenomenon could not be conclusively determined from our present data. One possibility is that PFL_3504 may mediate DHA efflux. Alternatively, this transporter may have an alternative substrate, and the accumulation of that substrate in the knockout mutant results in decreased expression of the *pchDHIEFKCBA* putative operon, hence indirectly impacting on secreted levels of DHA.

## Supporting Information

S1 Fig **Transmembrane topology predictions of the transporters of interest performed with HMMTOP version 2.0 for (A) *pchH*, (B) *fetF* and (C) PFL_3504.** Visuals were generated using TMRPres2D (Spyropoulos *et al*., 2004).
(TIF)

S2 Fig Confirmation of transporter gene truncations with PCR. Amplifications on gene of interests were performed using primers (A) PFL_3495-SK-F/R, (B) PFL_3503-SK-F/R and (C) PFL_3504-SK-F/R. PCR products were run on 1% TBE agarose gel. M: marker; -ve: negative control; WT: P. protegens Pf-5 wild-type; ΔpchH: P. protegens ΔpchH strain containing 1209 bp truncation within pchH gene; ΔfetF: P. protegens ΔfetF strain containing 800 bp truncation within fetF gene; Δ3504: P. protegens Δ3504 strain containing 930 bp truncation within PFL_3504 gene.
(TIF)

S3 Fig Optical densities depicting pyoverdine levels in supernatants derived from P. protegens strains as measured at 403 nm. Error bars represent standard deviations between three biological replicates.
(TIF)

S4 Fig **The profiles of Phenotype Microarray of *P*. *protegens* wild-type (WT) versus mutant strains (ΔpchH, ΔfetF and Δ3504) as tested using PM plates 09-13B (A), 14A-18C (B) and 19-20B (C).** The figure depicts wells from the microarray plates (PM09-20) where red and green areas represent the respiratory kinetics for wild-type and mutant strains respectively, while yellow areas indicate overlapping respiratory kinetics between the wild-type and mutant strains. The identity of the compound tested in each well can be found at the manufacturer’s website (http://www.biolog.com). Wells in which differential growth of Pf-5 vs. the mutant were observed are boxed in dark, as determined by the Omnilog software (summarized in S5 Table). Independent confirmatory tests were performed for the substrates in the boxed wells and the results are shown in Table 2.
(PDF)

S1 Table Primers for allelic exchange mutagenesis of transporter genes. (DOCX)

S2 Table Primers for verification of transporter gene truncations. (DOCX)

S3 Table Primers for construction of complementary plasmids. (DOCX)

S4 Table Primers in qRT-PCR experiments. (DOCX)

S5 Table Tests in Phenotypes microarray that showed differential respiratory kinetics between wild-type and mutant strains. (XLSX)
