# Supplementary figures and images for: Disruption of Transporters Affiliated with Enantio-Pyochelin Biosynthesis Gene Cluster of Pseudomonas protegens Pf-5 Has Pleiotropic Effects
Source: PLoS One. 2016 Jul 21;11(7):e0159884. doi: 10.1371/journal.pone.0159884 (PMC4956303; doi:10.1371/journal.pone.0159884)

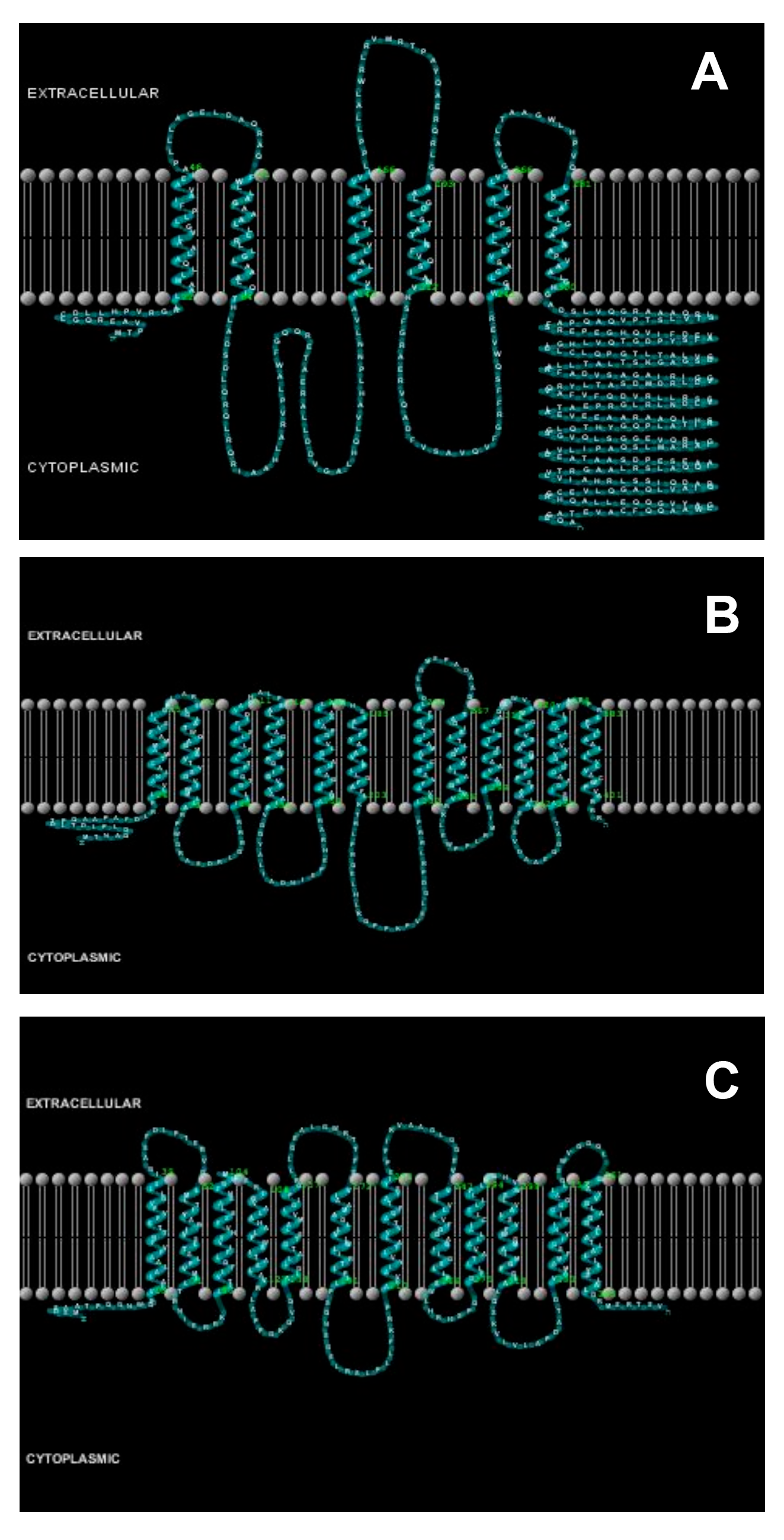

Supplement: S1 Fig — Transmembrane topology predictions of the transporters of interest performed with HMMTOP version 2.0 for (A) pchH, (B) fetF and (C) PFL_3504. Visuals were generated using TMRPres2D (Spyropoulos et al., 2004). (TIF) [file pone.0159884.s001.tif]

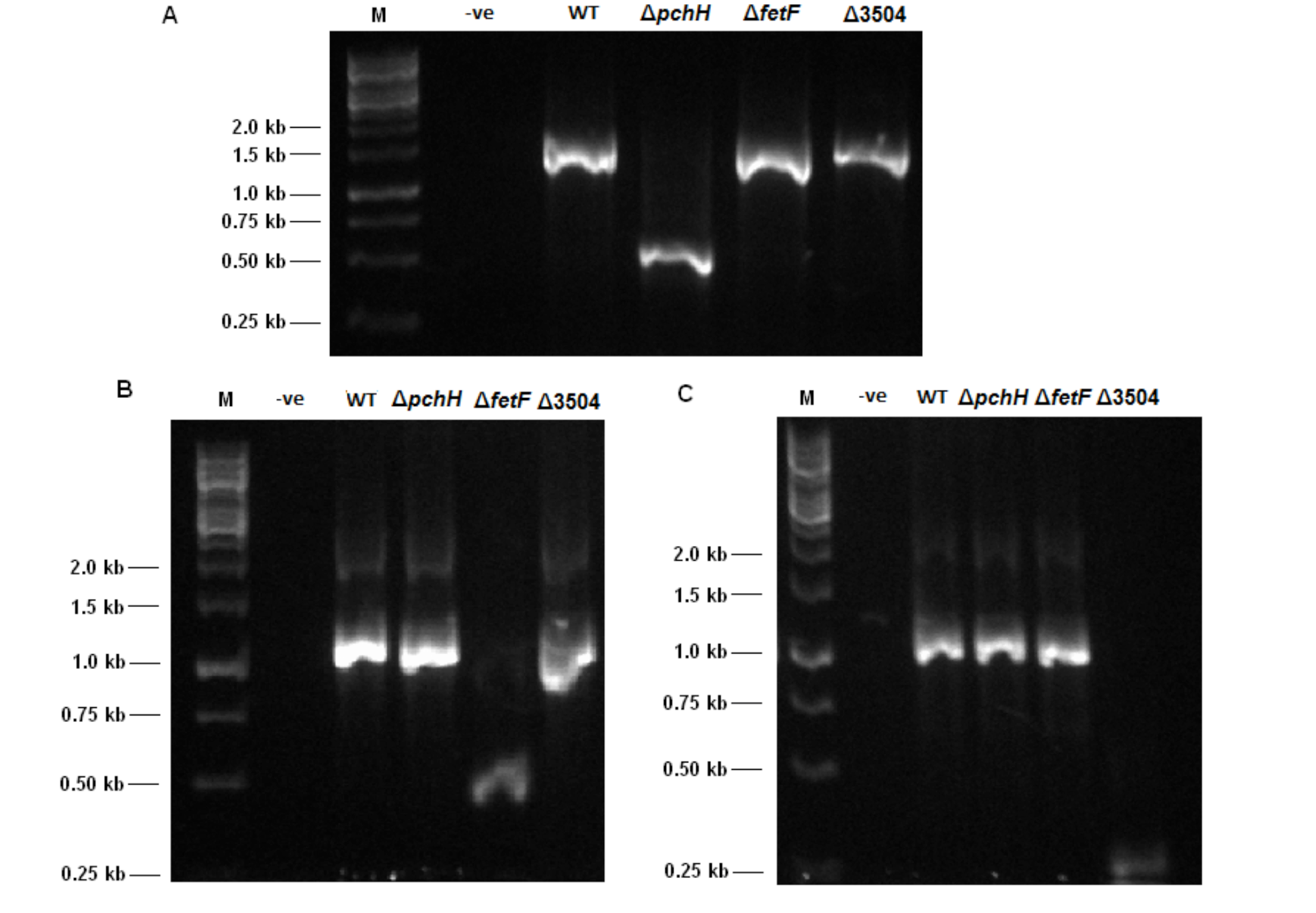

Supplement: S2 Fig — Amplifications on gene of interests were performed using primers (A) PFL_3495-SK-F/R, (B) PFL_3503-SK-F/R and (C) PFL_3504-SK-F/R. PCR products were run on 1% TBE agarose gel. M: marker; -ve: negative control; WT: P. protegens Pf-5 wild-type; ΔpchH: P. protegens ΔpchH strain containing 1209 bp truncation within pchH gene; ΔfetF: P. protegens ΔfetF strain containing 800 bp truncation within fetF gene; Δ3504: P. protegens Δ3504 strain containing 930 bp truncation within PFL_3504 gene. (TIF) [file pone.0159884.s002.tif]

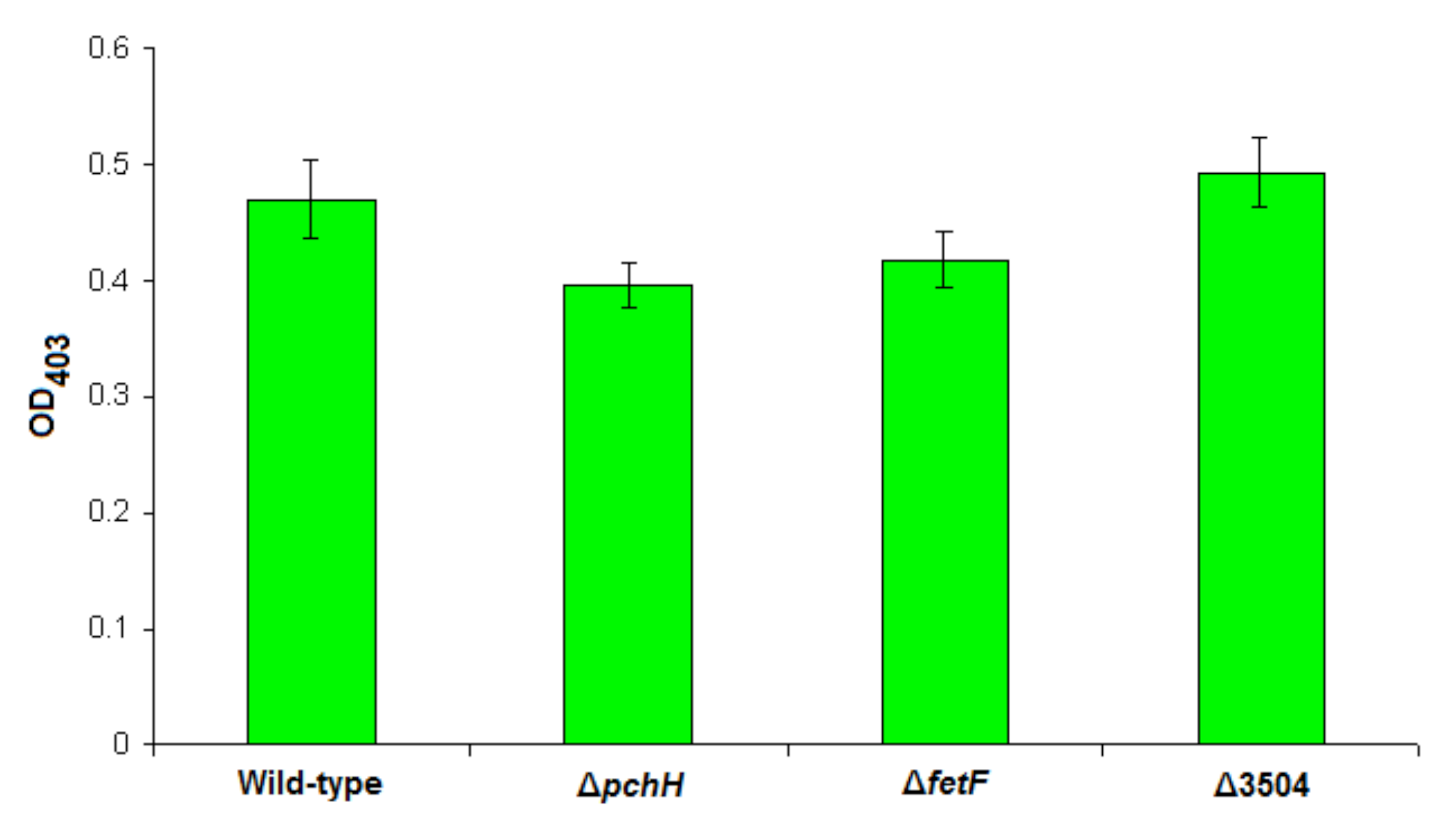

Supplement: S3 Fig — Error bars represent standard deviations between three biological replicates. (TIF) [file pone.0159884.s003.tif]
