# Supplementary material for: Disruption of Transporters Affiliated with Enantio-Pyochelin Biosynthesis Gene Cluster of Pseudomonas protegens Pf-5 Has Pleiotropic Effects
Source: PLoS One. 2016 Jul 21;11(7):e0159884. doi: 10.1371/journal.pone.0159884 (PMC4956303; doi:10.1371/journal.pone.0159884)
Supplement: S4 Fig — The profiles of Phenotype Microarray of P. protegens wild-type (WT) versus mutant strains (ΔpchH, ΔfetF and Δ3504) as tested using PM plates 09-13B (A), 14A-18C (B) and 19-20B (C). The figure depicts wells from the microarray plates (PM09-20) where red and green areas represent the respiratory kinetics for wild-type and mutant strains respectively, while yellow areas indicate overlapping respiratory kinetics between the wild-type and mutant strains. The identity of the compound tested in each well can be found at the manufacturer’s website (http://www.biolog.com). Wells in which differential growth of Pf-5 vs. the mutant were observed are boxed in dark, as determined by the Omnilog software (summarized in S5 Table). Independent confirmatory tests were performed for the substrates in the boxed wells and the results are shown in Table 2. (PDF) [file pone.0159884.s004.pdf]

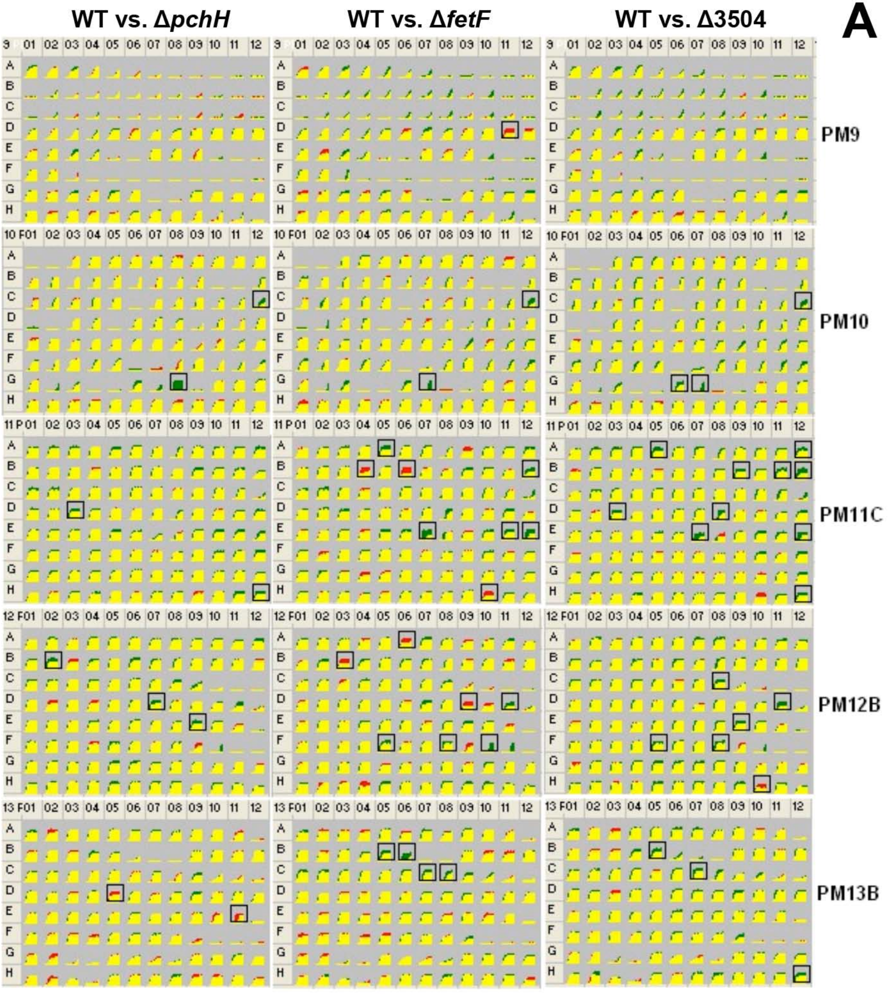
WT vs. ΔpchH
WT vs. ΔfetF
WT vs. Δ3504
A
PM9
PM10
PM11C
PM12B
PM13B

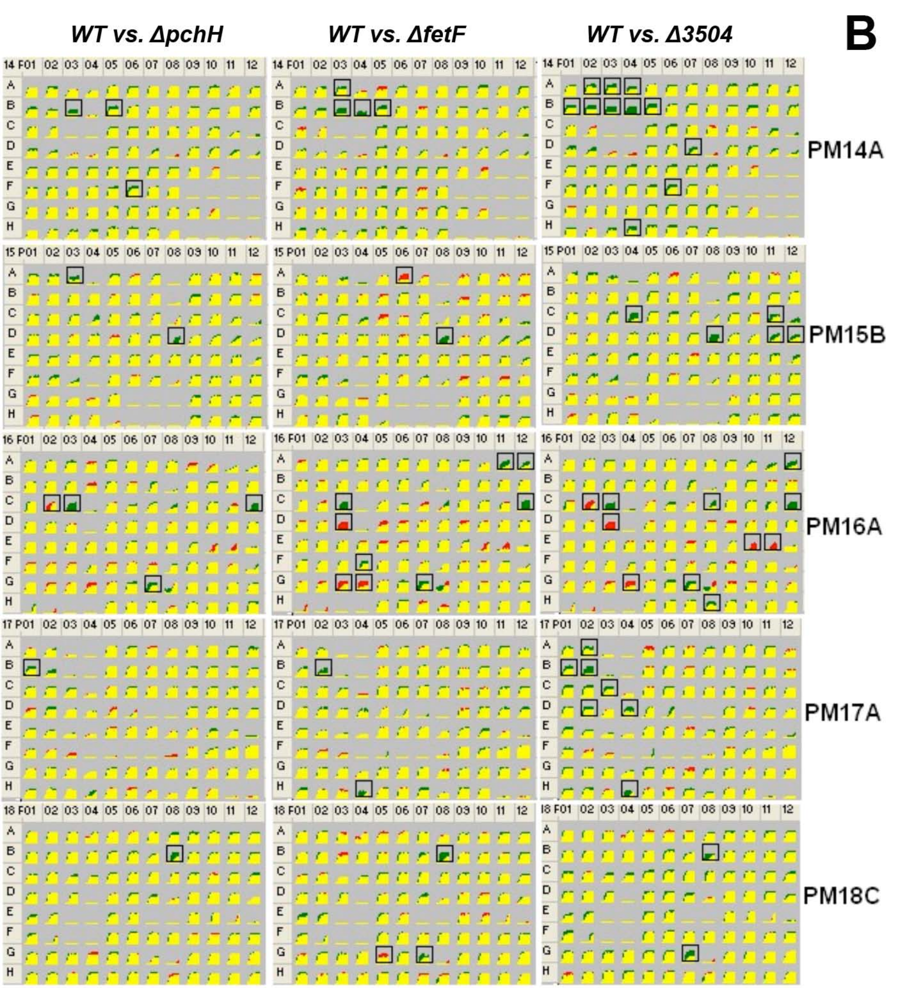
B
WT vs. ΔpchH
WT vs. ΔfetF
WT vs. Δ3504
PM14A
PM15B
PM16A
PM17A
PM18C

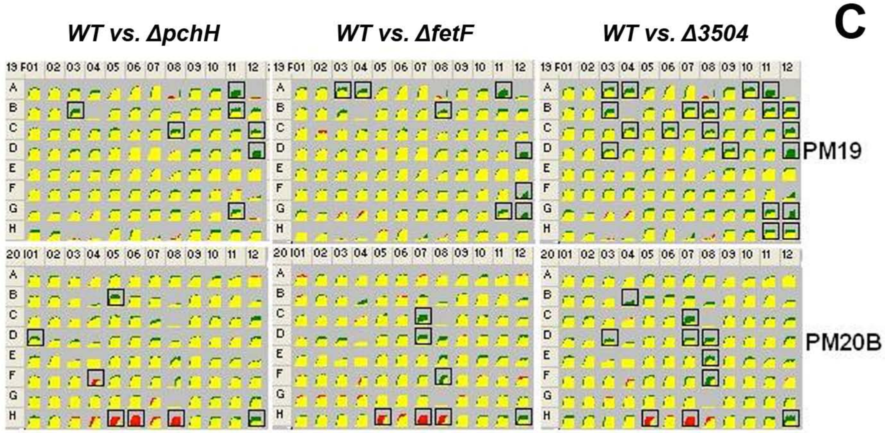

C
WT vs. ΔpchH
WT vs. ΔfetF
WT vs. Δ3504
PM19
PM20B
