# Supplementary material for: Disruption of Transporters Affiliated with Enantio-Pyochelin Biosynthesis Gene Cluster of Pseudomonas protegens Pf-5 Has Pleiotropic Effects
Source: PLoS One. 2016 Jul 21;11(7):e0159884. doi: 10.1371/journal.pone.0159884 (PMC4956303; doi:10.1371/journal.pone.0159884)
Supplement: S1 Table — (DOCX) [file pone.0159884.s005.docx]

**S1 Table: Primers for allelic exchange mutagenesis of transporter genes**

| **Genes** | **Amplifi-cation regions** | **Primer names** | **Primer sequences (5’-3’)*** |
| --- | --- | --- | --- |
| *pchH* | 5’ | PFL_3495-UpF  PFL_3495-NF-UpR | GTCAGTACACAAGCTTGACCTGATCAACCGCGGGGG  GTGCCTGGCTGAGGTGGGTGATCTGCAATGCC |
|  | 3’ | PFL_3495-NF-DownF  PFL_3495-DownR | GCAGATCACCCACCTCAGCCAGGCACCGATCC  GCTAGTACGAGGATCCCCGATGCGCAGGTTCAACGAC |
| *fetF* | 5’ | PFL_3503-UpF  PFL_3503-NF-UpR | GTCGACTACAAAGCTTGAACAAGTGCTGCAGGACCTGGAC  GAATGCTGGTGACAAAGGAACGCCATCATCGCCATG |
|  | 3’ | PFL_3503-NF-DownF  PFL_3503-DownR | GCGATGATGGCGTTCCTTTGTCACCAGCATTCTCGC  GTCATCGATCGGATCCGCAGTTGGGCTTTGCCTAT |
| PFL_3504 | 5’ | PFL_3504-UpF  PFL_3504-NF-UpR | GTCAGTTACGAAGCTTCAAGAGCCCTGCGCCTACGTC  GATGCCGGAGAAGGTGTTGAAGACAAAGGCCGCC |
|  | 3’ | PFL_3504-NF-DownF  PFL_3504-DownR | GCCTTTGTCTTCAACACCTTCTCCGGCATCTACAACATCG  CGTCGACTATGGATCCCCAGCAACTCGGTGGACG |

* Underlined nucleotides indicate restriction digestion sites
