# Supplementary material for: Disruption of Transporters Affiliated with Enantio-Pyochelin Biosynthesis Gene Cluster of Pseudomonas protegens Pf-5 Has Pleiotropic Effects
Source: PLoS One. 2016 Jul 21;11(7):e0159884. doi: 10.1371/journal.pone.0159884 (PMC4956303; doi:10.1371/journal.pone.0159884)
Supplement: S2 Table — (DOCX) [file pone.0159884.s006.docx]

**S2 Table: Primers for verification of transporter gene truncations**

| **Genes** | **Functions** | **Primer names** | **Primer sequences (5’-3’)** |
| --- | --- | --- | --- |
| *pchH* | Verification of Δ*pchH* construct | PFL_3495-SK-F  PFL_3495-SK-R | GGAATTCATAAGGAGGTAACTTAAATGACGCCCGTGGCTGAACG  CGGATCCTTAATGGTGATGATGGTGATGCGATCCTCGTGCCTGCTCCCCCGCC |
| *fetF* | Verification of Δ*fetF* construct | PFL_3503-SK-F  PFL_3503-SK-R | GGAATTCATAAGGAGGTAACTTAAATGACCAACGCTCAACAGCTGCCC  CGGATCCTTAATGGTGATGATGGTGATGCGATCCTCGGCGTCGGGCTA CGGGC |
| PFL_3504 | Verification of ΔPFL_3504 construct | PFL_3504-SK-F  PFL_3504-SK-R | GGAATTCATAAGGAGGTAACTTAAATGAGTGATTCCGCGGCGACACC  CGGATCCTTAATGGTGATGATGGTGATGCGATCCTCGGCTGGTGCGGCTCATGC |
