# Supplementary material for: Disruption of Transporters Affiliated with Enantio-Pyochelin Biosynthesis Gene Cluster of Pseudomonas protegens Pf-5 Has Pleiotropic Effects
Source: PLoS One. 2016 Jul 21;11(7):e0159884. doi: 10.1371/journal.pone.0159884 (PMC4956303; doi:10.1371/journal.pone.0159884)
Supplement: S3 Table — (DOCX) [file pone.0159884.s007.docx]

**S3 Table. Primers for construction of complementary plasmids**

| **Amplification products** | **Primer names** | **Primer sequences (5’-3’)*** |
| --- | --- | --- |
| Upstream region of *pchD* | UP3496-F  UP3496-R | AGCTAAGCTTGGCGAACTCCCTGTGG  GGAATTCGCTGTCTCCTGATGTTTTTTACG |
| *pchH* gene | 3495-pB-F  3495-pB-R | GGAATTCATGACGCCCGTGGCTGAACG  TGGATCCTCATGCCTGCTCCCCCGC |
| Upstream region of *fetA* | UP3498-F  UP3498-R | AGCTAAGCTTGCCCGCTCCTGCTCGAG  GGAATTCGCTTCACTCCGTGATGGGG |
| *fetF* gene | 3503-pB-F  3503-pB-R | GGAATTC ATGACCAACGCTCAACAGCT  TGGATCCCGTGTAGCCGCTGCCGCAGG |
| PFL_3504 gene with promoter and terminator regions | 3504-pB-F  3504-pB-R | AGCTAAGCTTGAAGCGGCGCTGCACCCTGG  GGAATTCCCTTCGGGCGAACGACG |

* Underlined nucleotides indicate restriction digestion sites
