# Supplementary material for: Disruption of Transporters Affiliated with Enantio-Pyochelin Biosynthesis Gene Cluster of Pseudomonas protegens Pf-5 Has Pleiotropic Effects
Source: PLoS One. 2016 Jul 21;11(7):e0159884. doi: 10.1371/journal.pone.0159884 (PMC4956303; doi:10.1371/journal.pone.0159884)
Supplement: S4 Table — (DOCX) [file pone.0159884.s008.docx]

**S4 Table. Primers in qRT-PCR experiments**

| **Loci** | **Gene products and annotated functions** | **Primer names** | **Primer sequences (5’-3’)** | **Product sizes (bp)** |
| --- | --- | --- | --- | --- |
| PFL_3488 | salicylate biosynthesis isochorismate synthase, PchA | PFL_3488-qF  PFL_3488-qR | CTGTTTGCCGCCAACCGCCA  GCACAGGTAGGCCCGCTTGG | 198 |
| PFL_3489 | isochorismate-pyruvate lyase, PchB | PFL_3489-qF  PFL_3489-qR | GGGCGGCAATGCTGTCCTCG  GACGACGTCCGCTGCGGTATC | 121 |
| PFL_3490 | enantio-pyochelin biosynthetic protein, PchC | PFL_3490-qF  PFL_3490-qR | CGTCGTTATCCGCAGCCGCA  GACCAGCTCGACATCCGCCG | 120 |
| PFL_3491 | saccharopine dehydrogenase, PchK | PFL_3491-qF  PFL_3491-qR | TTGTTCCAGGCAGGCCGTCG  AGCGTCGCAGCCTTCTGTGC | 96 |
| PFL_3492 | enantio-pyochelin synthetase F, PchF | PFL_3492-qF  PFL_3492-qR | AAGGCGCGGCAGTCTGGTTC  TGGGACATCCGCGAAGGGGT | 178 |
| PFL_3493 | enantio-pyochelin synthetase E, PchE | PFL_3493-qF  PFL_3493-qR | GCCAGCAGATGAGGCACGCA  CTGGAGCGCCAGCCAGCATT | 84 |
| PFL_3494 | ABC transporter, permease/ATP-binding protein, putative, PchI | PFL_3494-qF  PFL_3494-qR | ACAGCTCGCGATAACGGCCC  CCCCAGCGCCGGACAGATTC | 101 |
| PFL_3495 | ABC transporter, ATP-binding/permease protein, PchH | PFL_3495-qF  PFL_3495-qR | TCGCCCATCGCCTGAGCAGT CACAGCCCGGCATACACCCC | 124 |
| PFL_3496 | salicyl-AMP ligase, PchD | PFL_3496-qF  PFL_3496-qR | GCACCCCGAGGCGGTAAAGG  CCAACTGGCCGCCGGCTTAT | 119 |
| PFL_3497 | regulatory protein, PchR | PFL_3497-qF  PFL_3497-qR | TTCCCAACCCGCAGCACACC  TTGAGCTGGCCGCTGAGCAC | 148 |
| PFL_3498 | TonB-dependent outermembrane enantio-pyochelin receptor, FetA | PFL_3498-qF  PFL_3498-qR | TTCGACCCCAGCACCCCCTT  TGCGGTTATCGGTTCGGGCG | 148 |
| PFL_3499 | PepSY-associated membrane protein, FetB | PFL_3499-qF  PFL_3499-qR | GCTGGTAAGCCGCCGATGCT  CATCTGCGCACCGACCTCCC | 159 |
| PFL_3500 | FeCT family iron-chelate ABC transporter periplasmic iron-chelate-binding protein, FetC | PFL_3500-qF  PFL_3500-qR | CCAAGTGGCTGCACCCGGAG  GCTGGCGGGAATCGGCATGA | 92 |
| PFL_3501 | FeCT family iron-chelate ABC transporterpermease, FetD | PFL_3501-qF  PFL_3501-qR | GGCGCTGCTGCAATTCGTCG  GCAGGGAGAAGGGCAGGCAC | 140 |
| PFL_3502 | FeCT family iron-chelate ABC transporter ATP-binding protein, FetE | PFL_3502-qF  PFL_3502-qR | GTCGACCTCAACCGCCTGCC  TTCGACCCCGTGCAACACCG | 183 |
| PFL_3503 | transporter, putative, FetF | PFL_3503-qF  PFL_3503-qR | CAGCATTCTCGCGGCCCAGG CCCGACCACTACCCCCAGGC | 178 |
| PFL_3504 | major facilitator family transporter | PFL_3504-qF  PFL_3504-qR | CAACATCGGCATTGGCGGCG  AACAGCAAGGCCAGGGTCGC | 111 |
| PFL_5586* | 30S ribosomal protein S7, RpsG | PFL_5586-qF  PFL_5586-qR | ACGCTCGGCAACGGCTTTCT  CGTCGCGTAGCAGCCAAACG | 114 |

* Adapted from Lim *et al*. (2012)
